# Comparison of learning curves and cross-over effects using articulating laparoscopic instruments versus standard laparoscopic instruments

**DOI:** 10.1007/s00464-026-12977-1

**Published:** 2026-06-19

**Authors:** Jean-Paul Bereuter, Maximiliano Emmanuel Ceron Malpica, Mark Enrik Geissler, Josephine Runge, Jasmin Hasanovic, Anne Selbmann, Rona Berit Geissler, Grit Krause-Jüttler, Alexander Kern, Jürgen Weitz, Marius Distler, Felix von Bechtolsheim, Jakob Dobroschke, Florian Oehme

**Affiliations:** 1 Department of Visceral, Thoracic and Vascular Surgery, Faculty of Medicine and University Hospital Carl Gustav Carus, TUD Dresden University of Technology, Fetscherstraße 74, 01307 Dresden, Germany; 2 Surgical Skills Lab Dresden, Faculty of Medicine and University Hospital Carl Gustav Carus, Technische Universität Dresden, Dresden, Germany; 3 Department of General, Visceral and Thoracic Surgery, Proctology, Helios Klinikum Pirna, Pirna, Germany; 4 Centre for Tactile Internet With Human-in-the-Loop (CeTI), TUD Dresden University of Technology, Dresden, Germany

**Keywords:** Minimally invasive surgery, Articulating instruments, Laparoscopic skill analysis

## Abstract

**Background:**

Minimally invasive surgery evolves continuously with novel tools complementing standard laparoscopic instruments (SLI). Articulating laparoscopic instruments (ALI) provide enhanced dexterity resembling robotic systems while remaining more affordable and accessible. However, comparative data on their learning curve and performance are limited. This study investigated ALI training and performance in a controlled simulator setting.

**Methods:**

Fifty-three medical students participated. After baseline measurement, all participants underwent the same modified Fundamentals of Laparoscopic Surgery (FLS) training curriculum but were randomized in either using ALI or SLI. Learning curves were tracked for the Peg transfer and suture and knot tasks until a predefined proficiency level was reached. All participants then completed final assessments using both instrument types. The primary endpoint was peak force; secondary endpoints included instrument motion metrics, task time, error rate, and SURG-TLX workload scores.

**Results:**

The ALI group demonstrated a slower learning curve in the Peg transfer task compared to the SLI group based on task time (*p* < 0.001), peak force (*p* = 0.02), and path length (*p* < 0.001). In the final assessment, peak force was comparable between groups (Peg: *p* = 0.17; Suture: *p* = 0.28), while ALI use resulted in greater path length (Peg: *p* < 0.001; Suture: *p* = 0.01) and longer task time (*p* < 0.001). Error rates and SURG-TLX scores did not differ. In the crossover assessment, prior ALI training resulted in comparable results for SLI for most parameters, including peak force (Peg: *p* = 0.97, suture: *p* = 0.05), instrument speed (Peg: *p* = 0.13, suture: *p* = 0.27) or error rate (Peg: *p* = 0.06; suture: *p* = 0.21).

**Conclusion:**

Although ALI require a longer learning curve, training with these instruments transfers effectively to standard laparoscopic skills. Their robotic-like motion and accessibility support incorporating ALI into surgical education.

**Graphical abstract:**

**Figure:**
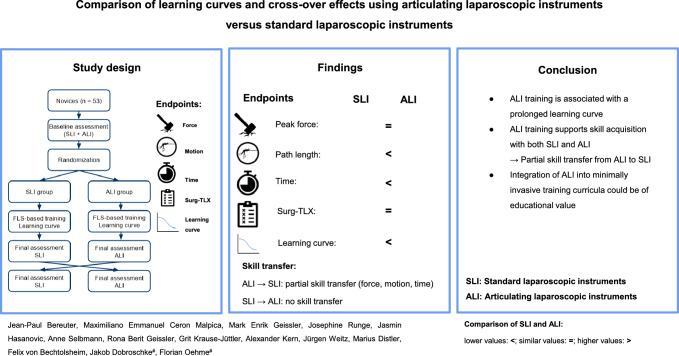

**Supplementary Information:**

The online version contains supplementary material available at 10.1007/s00464-026-12977-1.

The use of standard laparoscopic instruments (SLI) presents inherent ergonomic and technical challenges, particularly the restriction of instrument movement imposed by the fixed entry point through the abdominal wall [1]. Due to limited degrees of freedom, increased task complexity can be observed, especially in confined spaces or when operating at challenging angles [2].

To address these limitations, articulating laparoscopic instruments (ALI) have been developed to provide an additional degree of freedom at the instrument tip [3, 4]. By enabling articulation within the abdominal cavity, these instruments aim to enhance access even to challenging areas while they mimic the dexterity of robotic surgery. Apart from their maneuverability, the lower cost may provide an accessible alternative to robotic systems [5, 6]. Despite their theoretical advantages, articulating instruments may also introduce drawbacks, such as increased mechanical complexity, altered tactile feedback, and a steeper learning curve compared with standard rigid laparoscopic instruments [7].

Previous studies have examined the role of articulating instruments in specific procedures. Articulating instruments have been shown to be feasible and safe, with comparable outcomes to conventional laparoscopy in upper and lower gastrointestinal surgery [8–11]. Moreover, they also show promising applications in single port approaches as well as more complex surgical procedures in both abdominal and thoracic surgery [12–16]. However, evidence comparing their learning curve as well as performance to conventional laparoscopy in controlled simulator environments remains limited.

The present study compares laparoscopic performance using articulating laparoscopic instruments versus standard laparoscopic instruments. We evaluated learning curves and laparoscopic skill based on tissue handling and motion parameters, task completion time and error rates to determine whether articulating instruments confer measurable performance advantages over conventional laparoscopic instruments.

## Material and methods

This single-center, prospective randomized, controlled, open-label study was approved by the ethics committee of the Technische Universität Dresden (EK285072016). Participants were informed about the study and gave written consent to participate. In addition, this study was conducted in accordance with the CONSORT (Consolidated Standards of Reporting Trials) statement [17].

### Participants

This study was conducted at the University Hospital Dresden between January 2025 and June 2025. The study cohort consisted of 53 medical students without previous experience in laparoscopic surgery. Participants were randomly allocated to either the standard laparoscopic instruments group (SLI group) or the articulating laparoscopic instruments group (ALI group).

### Randomization

After enrollment, all students participated in a baseline training course where they received theoretical information about both instrument classes and the conducted tasks. In addition, they performed one Peg transfer task using both SLI and ALI to determine baseline performance. Based on the average peak force applied for both instrument classes, participants were randomly allocated to either the SLI or the ALI training group utilizing block randomization.

### Training curriculum

**Fig. 1:**
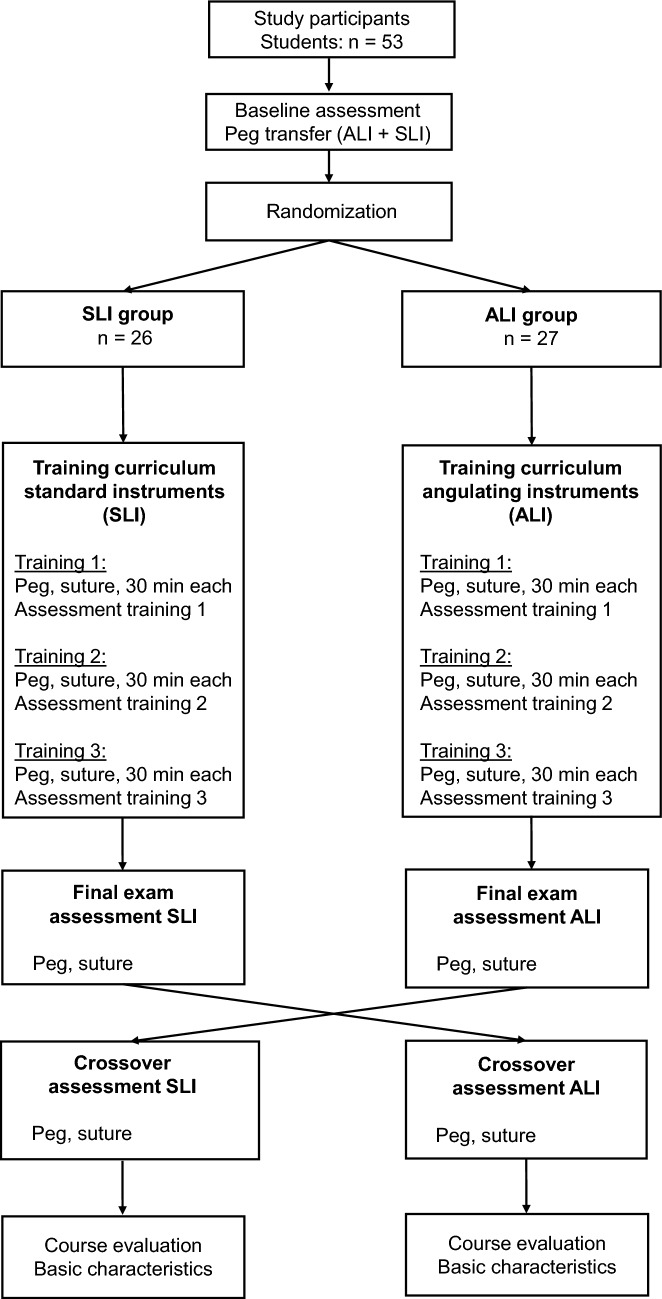
Flow chart representing an overview of the conducted study

After randomization, all students were trained according to a standardized modified Fundamentals of Laparoscopic Surgery (FLS) training curriculum [18]. Participants of the SLI group were trained using standard laparoscopic instruments (SLI; laparoscopic forceps and needle holder, Karl Storz), while participants of the ALI group were trained using articulating laparoscopic instruments (ALI; Artisential instruments: Maryland dissectors and needle holders, Livsmed). During the curriculum, all participants had to perform two laparoscopic tasks: the peg transfer and surgical suture and knot task. The conducted training curriculum consisted of 3 practical training sessions, where participants were allowed to train 30 min per task. An overview of the study and the conducted training curriculum can be seen in Fig. 1.

### Setting and measurements

The measurements were conducted in the surgical skills lab at the University Hospital Dresden. Surgical performance in terms of force exertion and motion parameters were measured using the ForceSense device (Medishield B.V., Delft, The Netherlands). The evaluated tasks were mounted on a platform attached to the ForceSense device within a standard laparoscopic box trainer. This system allows for independent measurement of the motion of two laparoscopic instruments and records the force exerted (in Newtons) on the task platforms.

To monitor the learning progress throughout the training curriculum, participants’ performance was assessed after each of the three practical training sessions, allowing for the recording of individual learning curves. As laparoscopic suturing was highly challenging and time consuming for novices, baseline measurements were obtained only for the Peg transfer task. Although both the Peg transfer and suture and knot tasks were assessed after each training session, learning curves were analyzed exclusively for the Peg transfer task due to the absence of baseline data for the suture and knot task. After completing the standardized training curriculum, participants from both the SLI and ALI groups were assessed in a final exam using the instrument type on which they had been trained. Additionally, a crossover assessment was conducted, wherein SLI group participants were evaluated using ALI, and ALI group participants were assessed with SLI.

### Primary endpoint

#### Laparoscopic performance based on force parameters

Key force metrics included parameters such as peak force and peak impulse. The measured parameters were already described by Hardon et al. and are defined as follows [18]:

Peak force [N]: The maximal force exerted during a task.

Peak impulse [Ns]: The maximum combined effect of force and duration during the task, calculated as the largest area under the force–time curve for a single force peak.

Peak force and impulse capture complementary aspects of mechanical loading and may therefore serve as surrogate markers of tissue stress during operative tasks. Although validated in vivo thresholds for safe values are lacking, both parameters have been consistently associated with tissue damage in experimental and dry-lab studies when elevated. Accordingly, they represent potential clinically relevant metrics for performance assessment, with lower values indicating a reduced mechanical burden on tissue and a potentially lower risk of intraoperative injury. Therefore, these metrics have been chosen for the primary endpoint in this study.

### Secondary endpoints

#### Laparoscopic performance based on motion parameters

Basic motion metrics such as mean instrument speed and path length. The motion parameters were measured separately for each hand. The measured parameters were already described by Hardon et al. and are defined as follows [18]:

Path length [mm]: The total distance traveled by the tip of the laparoscopic instruments during a task.

Instrument speed [cm/s]: The average rate of motion of the laparoscopic instruments over the course of the conducted task.

#### Laparoscopic performance based on task completion time, surgical errors and workload

The task completion time as well as the number of surgical errors were recorded. In addition, significant errors were defined for each task and recorded during each measurement based on previous studies [19, 20]. Error occurrences across all tasks were recorded by a single blinded observer to ensure objectivity. An overview of the utilized error definition for each task is shown in Supplementary Table 1.

Errors were recorded categorically, with higher values indicating greater deviation from the state of the art performance. Peg transfer was scored depending on the number of triangles dropped (0 no triangle dropped, 1 triangles dropped). In the suture and knot task, performance was assessed for accuracy, adaptation, and tightness, each scored 0 to 2 based on predefined criteria. To assess the participants’ workload in this study, we selected the Surgery Task Load Index (SURG-TLX) as a secondary endpoint [21]. This questionnaire is a validated and procedure-specific adaptation of the existing NASA Task Load Index (NASA-TLX) designed to evaluate cognitive and environmental demands of the operating room. The SURG-TLX incorporates six dimensions particularly relevant to surgical performance: mental demands, physical demands, temporal demands, task complexity, situational stress and intraoperative distractions. Each dimension is rated by the participants on a Likert scale and a weighted composite score is subsequently calculated based on pairwise comparisons of the relative importance of each dimension. Finally, an overall workload index can be generated out of weighted composite scores. The multidimensional structure of the SURG-TLX allows for a comprehensive assessment of both intrinsic task difficulty and extrinsic stressors, making it suitable for evaluating the participants’ workload in our study.

### Statistical analysis

Statistical analyses were performed using SPSS version 30 (IBM Corp., Armonk, NY, USA) and GraphPad Prism version 10.5 (GraphPad Software, San Diego, CA, USA). Normality of continuous data was assessed using the Kolmogorov–Smirnov test and inspection of frequency distributions. Participant characteristics are presented as mean ± standard deviation (SD) for continuous variables, and as frequencies for categorical variables. Depending on distribution and variance, comparisons between conditions were conducted using Welch’s t test, paired t test, or the Wilcoxon signed-rank test. Learning curves (LCs) were generated in Prism. Grouped LCs were compared using two-way ANOVA. No missing data were observed for the primary analysis. Statistical significance was set at *p* < 0.05. Unless otherwise stated, values reported in the results section represent medians.

## Results

### Basic participant characteristics

Fifty-three medical students participated in this single-center prospective randomized crossover trial. After baseline assessment and subsequent randomization, 26 participants were allocated to the SLI group and 27 to the ALI group. The basic participant characteristics of the cohort are summarized in Table 1. Overall, no significant differences were found between both groups concerning age (SLI: 25 years vs. ALI: 24.7 years, *p* = 0.82), the distribution of sex (SLI: 50% male vs. ALI: 67% male, *p* = 0.22), dominant hand (= DH, SLI: 89% right vs. ALI: 85% right, *p* = 0.73) and academic year (SLI: 5th AY vs. ALI: 5th AY, *p* = 0.76).

**Table 1:** Basic participant characteristics (*n* = 53). Values are depicted as median and IQR or number and %

Items	SLI group (n = 26)	ASI group (*n* = 27)	*p*-value
Age, median (IQR)	25 (22—28)	25 (22—27)	0.82
Sex, *n* (%)			0.22
Male	13 (50)	18 (67)	
Female	13 (50)	9 (33)	
DH, *n* (%)			0.73
Right	23 (88)	23 (85)	
Left	3 (12)	4 (15)	
AY			0.76
1	4 (15)	3 (10)	
2	5 (19)	8 (30)	
3	3 (12)	5 (19)	
4	5 (19)	3 (11)	
5	9 (35)	8 (30)	

*DH* dominant hand, *AY* academic year

### Learning curve for SLI versus ALI

**Fig. 2:**
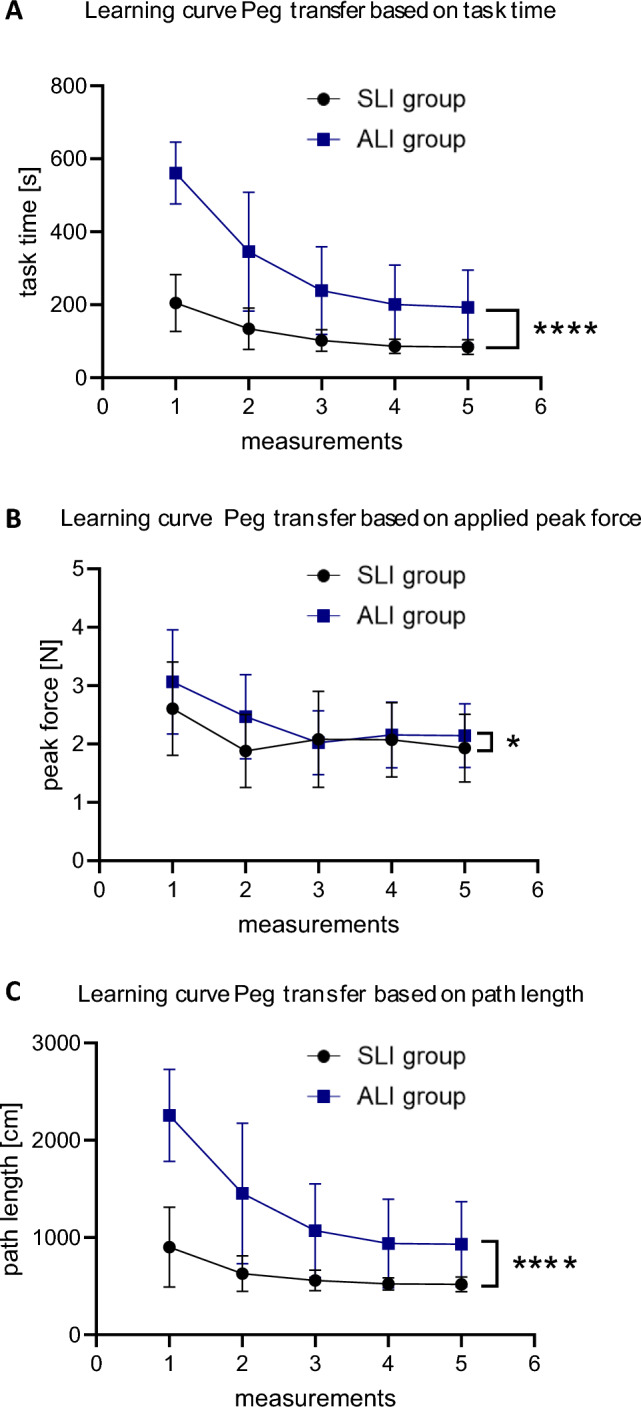
Learning curves (LCs) for the Peg transfer task based on task time, peak force and path length. Combined LCs were compared using two-way ANOVA

To generate learning curves, five performance different assessments were conducted throughout the study. Measurement 1 represents the baseline assessment prior to the start of the training. Measurements 2 to 4 were performed sequentially after each of the three conducted training sessions, reflecting the progress during the curriculum. Measurement 5 represents the final assessment conducted upon completion of the training curriculum. When analyzing the learning curves (LCs) for the Peg transfer task, significant differences between the SLI and the ALI groups were found (see Fig. 2). For the applied peak force, the ALI group showed a differing learning progress with significantly higher peak forces throughout the training curriculum (*p* = 0.02). In addition, participants in the ALI group required significantly more task time across all stages of training and plateaued at higher values, as confirmed by ANOVA (*p* < 0.001). This pattern was not limited to task time but extended to other performance parameters. A similar effect was observed for path length, where participants in the ALI group showed a more prolonged learning trajectory (*p* < 0.001).

### Primary endpoint: analysis of force parameters

#### Final exam assessment – Peg transfer task

**Fig. 3:**
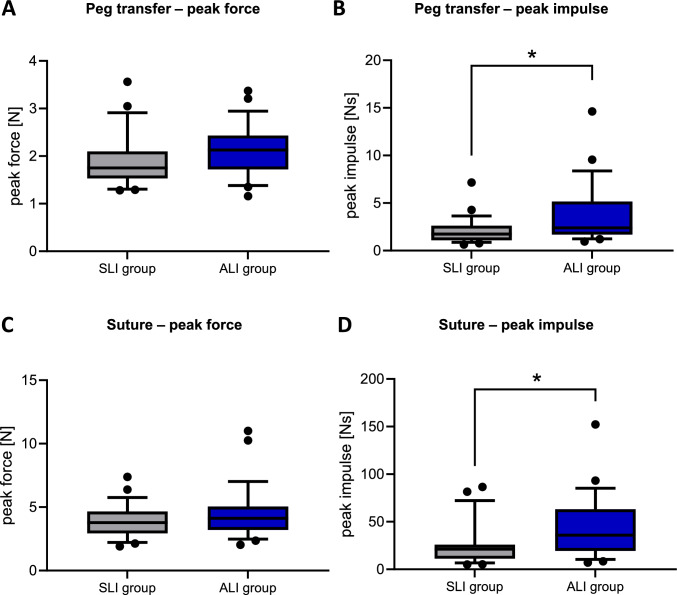
Comparison of force parameters between the SLI and ALI groups for the Peg transfer and the suture and knot task during the final exam. All graphs show mean values and standard deviations. *p < 0.05, **p < 0.01, ***p < 0.001

As shown in Fig. 3, participants of the SLI group applied a similar peak force in the Peg transfer task compared to those of the ALI group (SLI group: 1.8 N vs. ALI group: 2.1 N, *p* = 0.17) during the final exam assessment. In contrast, participants of the SLI group applied a significantly lower peak impulse than those of the ALI group (SLI group: 1.7 Ns vs. ALI group: 2.4 Ns, *p* = 0.015).

#### Final exam assessment – suture and knot task

During the final exam assessment, participants of both groups applied a similar peak force (SLI group: 3.8 N vs. ALI group: 4.1 N, *p* = 0.28) in the suture and knot task (see Fig. 3). However, participants of the SLI group applied a significantly lower peak impulse compared to participants of the ALI group (SLI group: 21.2 Ns vs. ALI group: 35.8 Ns, *p* = 0.028).

#### Crossover assessment Peg transfer task

When including the crossover assessment, where participants of the SLI group used ALI and participants of the ALI group used SLI, the following pattern was observed (see Supplementary Fig. 1): Participants of the SLI group applied a significantly higher peak force in the Peg transfer task when using ALI compared to SLI (SLI: 1.75 N vs. ALI: 2.8 N, *p* < 0.001). Similarly, regarding the applied peak impulse, participants of the SLI group showed significantly higher values when using ALI compared to SLI (SLI: 1.7 Ns vs. ALI: 5.6 Ns, *p* < 0.001). Interestingly, participants of the ALI group showed similar values between SLI and ALI in the crossover assessment of the final exam. Values for the applied peak force (SLI: 1.8 N vs. ALI: 2.1 N, *p* = 0.22) and peak impulse (SLI: 2.5 Ns vs. ALI: 2.4 Ns, *p* = 0.2) were not significantly different between both used instrument types. When comparing the use of SLI between the SLI and the ALI group, no significant differences were observed regarding peak force (SLI: 1.8 N vs. ALI: 1.8 N, *p* = 0.97) and peak impulse (SLI: 1.7 Ns vs. ALI: 2.5 Ns, *p* = 0.1). However, when comparing the use of ALI between the SLI and the ALI group, significant differences were found in peak force (SLI: 2.8 N vs. ALI: 2.1 N, *p* = 0.004) and peak impulse (SLI: 5.6 Ns vs. ALI: 2.4 Ns, *p* = 0.03).

#### Crossover assessment suture and knot task

The results for the crossover assessment for the suture and knot task can be also seen in Supplementary Fig. 1: Participants of the SLI group applied a significantly higher peak force in the suture and knot transfer when using ALI compared to SLI (SLI: 3.8 N vs. ALI: 4.1 N, *p* = 0.04). Similarly, regarding the applied peak impulse, participants of the SLI group showed significantly higher values when using ALI compared to SLI (SLI: 21.2 Ns vs. ALI: 58.4 Ns, *p* = 0.003). Interestingly, participants of the ALI group were significantly better when using SLI in the crossover assessment compared to ALI in terms of peak impulse (SLI: 20.7 Ns vs. ALI: 35.8 Ns, *p* = 0.015), while the applied peak force was comparable between both used instruments (SLI: 4.1 N vs. ALI: 4.4 N, *p* = 0.44).

When comparing the use of SLI between the SLI and the ALI group, no significant differences were observed regarding peak force (SLI: 3.8 N vs. ALI: 4.4 N, *p* = 0.05) and peak impulse (SLI: 21.2 Ns vs. ALI: 20.7 Ns, *p* = 0.95). However, when comparing the use of ALI between the SLI and the ALI group, significant differences were observed in the applied peak impulse (SLI: 58.4 Ns vs. ALI: 35.8 Ns, *p* = 0.04), while the peak force showed no differences (SLI: 4.1 N vs. ALI: 4.1 N, *p* = 0.47).

### Secondary endpoints: comparison of motion parameters between SLI and ALI

#### Final exam assessment – Peg transfer task

**Fig. 4:**
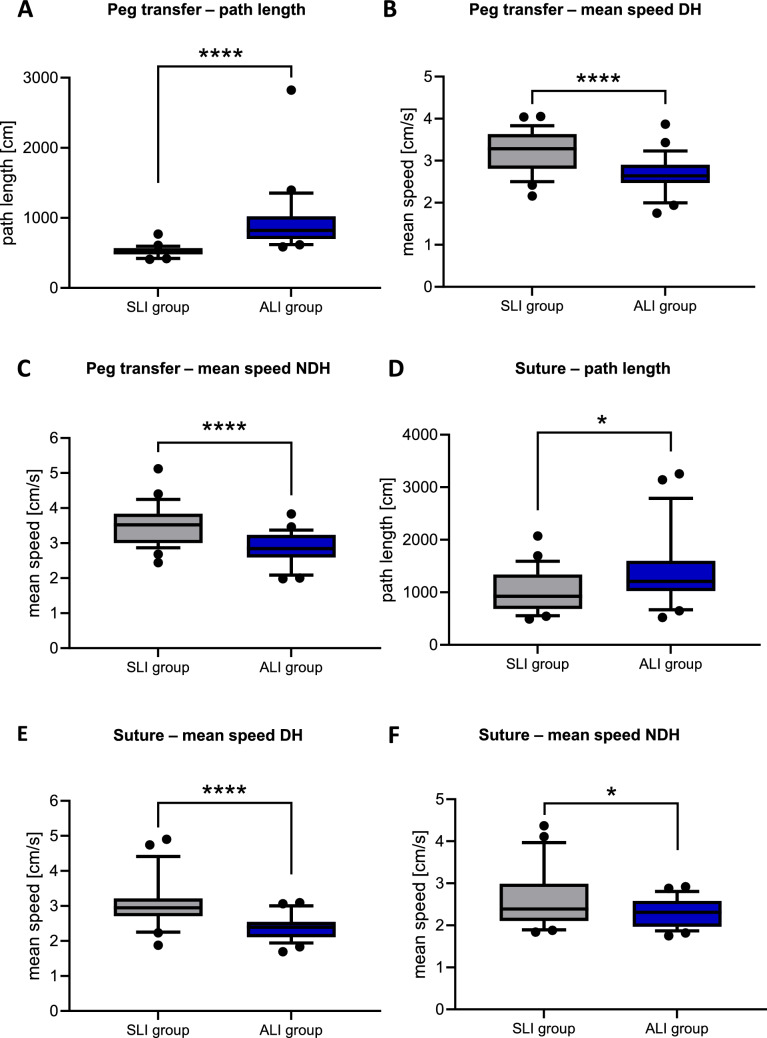
Comparison of motion parameters between the SLI and ALI groups for the Peg transfer and the suture and knot task during the final exam. All graphs show mean values and standard deviations. *p < 0.05, **p < 0.01, ***p < 0.001

As shown in Fig. 4, participants of the SLI group used a significantly lower total path length compared to those of the ALI group in the Peg transfer task (SLI group: 504 cm vs. ALI group: 822 cm, *p* < 0.001) during the final exam assessment. In line with this, participants of the SLI group also used a significantly higher instrument speed compared to those of the ALI group for the dominant hand (DH: SLI group: 3.3 cm/s vs. ALI group: 2.6 cm/s, *p* < 0.001) and the non-dominant hand (NDH: SLI group: 3.5 cm/s vs. ALI group: 2.8 cm/s, *p* < 0.001).

#### Final exam assessment – suture and knot task

Similar results were observed for the suture and knot task (see Fig. 4). During the final exam assessment, participants of the SLI group used a significantly lower total path length compared to those of the ALI group (SLI group: 926 cm vs. ALI group: 1207 cm, *p* = 0.01). In line with this, participants of the SLI group also used a significantly higher instrument speed compared to those of the ALI group for the dominant hand (DH: SLI group: 3 cm/s vs. ALI group: 2.4 cm/s, *p* < 0.001) and the non-dominant hand (NDH: SLI group: 2.4 cm/s vs. ALI group: 2.3 cm/s, *p* = 0.036).

#### Crossover assessment Peg transfer task

The results for the crossover assessment for the Peg transfer task can be found in Supplementary Fig. 2: Participants of the SLI group used significantly more path length when using ALI compared to SLI (SLI: 504 cm vs. ALI: 1613 cm, *p* < 0.001). Moreover, participants of the SLI group applied significantly higher instrument speed of the DH (SLI: 3.3 cm/s vs. ALI: 2.3 cm/s, *p* < 0.001) and NDH (SLI: 3.5 cm/s vs. ALI: 2.4 cm/s, *p* < 0.001) when using SLI compared to ALI. Similarly, participants of the ALI group used significantly more path length when using ALI compared to SLI (SI: 638 cm vs. AI: 822 cm, *p* = 0.001). Concerning instrument speed, participants of the ALI group also showed faster instrument speed when using SLI compared to ALI for the DH (SLI: 3 cm/s vs. ALI: 2.6 cm, *p* < 0.001) and the NDH (SLI: 3.4 cm/s vs. ALI: 2.8 cm, *p* < 0.001).

When comparing the use of SLI between the SLI and the ALI group, no difference was observed for the use of SLI concerning instrument speed of the DH (SLI: 3.3 cm/s vs. ALI: 3 cm/s, *p* = 0.13) and NDH (SLI: 3.3 cm/s vs. ALI: 3 cm/s, *p* = 0.13). Interestingly, when considering the used path length, participants of the ALI group used significantly more path length when using SLI (SLI: 504 cm vs. ALI: 638 cm, *p* < 0.001). When comparing the use of ALI between the SLI and the ALI group, significant differences were seen in path length (SLI: 1613 cm vs. ALI: 822 cm, *p* < 0.001), instrument speed of the DH (SLI: 2.3 cm/s vs. ALI: 2.6 cm/s, *p* = 0.004) and NDH (SLI: 2.4 cm/s vs. ALI: 2.8 cm/s, *p* = 0.007).

#### Crossover assessment suture and knot task

The results for the crossover assessment for the suture and knot task can be also seen in Supplementary Fig. 2: Participants of the SLI group used significantly more path length when using ALI compared to SLI (SLI: 926 cm vs. ALI: 2201 cm, *p* < 0.001). Moreover, participants of the SLI group applied significantly higher instrument speed of the DH (SLI: 3 cm/s vs. ALI: 2.2 cm/s, *p* < 0.001) and NDH (SLI: 2.4 cm/s vs. ALI: 2.3 cm/s, *p* = 0.04) when using SLI compared to ALI. Similarly, participants of the ALI group used significantly more path length when using ALI compared to SLI (SLI: 1048 cm vs. ALI: 1207 cm, *p* = 0.001). Concerning instrument speed, participants of the ALI group also showed faster instrument speed when using SLI compared to ALI for the DH (SLI: 2.9 cm/s vs. ALI: 2.4 cm, *p* < 0.001), while no difference was observed for the NDH (SLI: 2.3 cm/s vs. ALI: 2.3 cm, *p* = 0.86).

When comparing the use of SLI between the SLI and the ALI group, no difference was observed for the use of SLI concerning path length (SLI: 926 cm vs. ALI: 1048 cm, *p* = 0.13), instrument speed of the DH (SLI: 3 cm/s vs. ALI: 2.9 cm/s, *p* = 0.27) and NDH (SLI: 2.4 cm/s vs. ALI: 2.3 cm/s, *p* = 0.06). Interestingly, when considering the used path length, participants of the ALI group used significantly more path length when using SLI (SLI: 504 cm vs. ALI: 638 cm, *p* < 0.001). When comparing the use of ALI between the SLI and the ALI group, a significant difference was seen for the path length (SLI: 2201 cm vs. ALI: 1207 cm, *p* < 0.001), while instrument speed of the DH (SLI: 2.2 cm/s vs. ALI: 2.4 cm/s, *p* = 0.11) and the NDH (SLI: 2.3 cm/s vs. ALI: 2.3 cm/s, *p* = 0.48) was comparable between both groups.

### Secondary endpoint: comparison of task completion time, error rates and workload between SLI and ALI

#### Final exam assessment – Peg transfer task

**Fig. 5:**
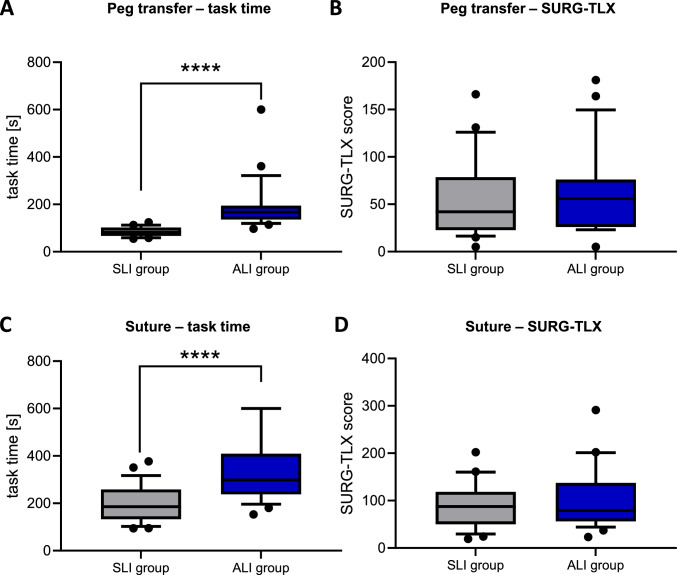
Comparison of task completion times and SURG-TLX scores between the SLI and ALI groups for the Peg transfer and the suture and knot task during the final exam. All graphs show mean values and standard deviations. *p < 0.05, **p < 0.01, ***p < 0.001

The results for the task completion times, error rates and the participants’ workload are shown in Fig. 5 and Table 2. Participants of the SLI group were significantly faster when conducting the Peg transfer task compared to participants of the ALI group (SLI group: 83 s vs. ALI group: 167 s, *p* < 0.001). In contrast, error rates (SLI group: 0 vs. ALI group: 0, *p* = 0.525) and SURG-TLX scores (SLI group: 42 vs. ALI group: 56, *p* = 0.35) were not significantly different between both groups in the final exam assessment.

**Table 2:** Comparison of error occurrence between the SLI and ALI groups for the Peg transfer and the suture and knot task during the final exam

Task	Error	SLI group	ALI group	*p*-value
Peg transfer task				0.525
	0	22 (84.6)	21 (77.8)	
	1	4 (15.4)	6 (22.2)	
*Suture and knot task*				
Accuracy				0.519
	0	10 (38.4)	7 (25.9)	
	1	8 (30.8)	8 (29.6)	
	2	8 (30.8)	12 (44.5)	
Adaptation				0.068
	0	23 (88.5)	20 (74.1)	
	1	3 (11.5)	2 (7.4)	
	2	0 (0)	5 (18.5)	
Tightness				0.548
	0	24 (92.3)	23 (85.2)	
	1	2 (7.7)	3 (11.1)	
	2	0 (0)	1 (3.7)	

#### Final exam assessment – suture and knot task

The results for the corresponding task completion times, error rates and the participants’ workload for the suture and knot task are also shown in Fig. 5 and Table 2. In line with the results of the Peg transfer task, participants of the SLI group again significantly faster than those of the ALI group (SLI group: 186 s vs. ALI group: 298 s, *p* < 0.001). However, error rates (accuracy: *p* = 0.519, adaptation: *p* = 0.068, tightness: *p* = 0.548) and SURG-TLX scores (SLI group: 88 vs. ALI group: 79, *p* = 0.37) remained comparable.

#### Crossover assessment Peg transfer task

The results for the crossover assessment for the Peg transfer task can be found in Supplementary Fig. 3 and Supplementary Table 2: Participants of the SLI group performed significantly faster when using SLI compared to ALI (SLI: 109 s vs. ALI: 413 s, *p* < 0.001). Moreover, the error rate (SLI: 0 vs. ALI: 1, *p* < 0.001) and the SURG-TLX score (SLI: 42 vs. ALI: 131, *p* < 0.001) were significantly higher in the SLI group when using ALI compared to SLI. Interestingly, participants of the ALI group were also significantly faster when using SLI compared to ALI (SLI: 83 s vs. ALI: 167 s, *p* < 0.001). Similarly, SURG-TLX scores were significantly higher when using ALI compared to SLI (SLI: 35 vs. ALI: 56, *p* = 0.007), while error rates were comparable between both instrument types used (SLI: 0 vs. ALI: 0, *p* = 0.148). When comparing the use of SLI between the SLI and the ALI group, a significant difference was observed for the task time (SLI: 83 s vs. ALI: 109 s, *p* < 0.001), while SURG-TLX scores (SLI: 42 vs. ALI: 35, *p* = 0.41) were similar between both groups. Regarding the use of ALI between the SLI and the ALI group, significant differences were observed for task time (SLI: 413 s vs. ALI: 167 s, *p* < 0.001) and SURG-TLX score (SLI: 131 vs. ALI: 56, *p* < 0.001).

#### Crossover assessment suture and knot task

The results for the crossover assessment for the suture and knot task can be also seen in Supplementary Fig. 3 and Supplementary Table 2: Participants of the SLI group were significantly faster when using SLI compared to ALI (SLI: 186 s vs. ALI: 600 s, *p* < 0.001). In addition, participants of the SLI group had a significantly higher error rate (accuracy: p = 0.039, adaptation: *p* = 0.001, tightness: *p* = 0.004) and SURG-TLX score (SLI: 88 vs. ALI: 166, *p* < 0.001) when using ALI compared to SLI. Participants of the ALI group showed a significantly longer task time (SLI: 231 s vs. ALI: 298 s, *p* = 0.005) and higher SURG-TLX score (SLI: 69 vs. ALI: 79, *p* = 0.01), while error rate was similar between the use of both instrument types (accuracy: *p* = 0.943, adaptation: 0.454, tightness: 0.236). When comparing the use of SLI between the SLI and the ALI group, significant differences were observed for the task time (SLI: 186 s vs. ALI: 231 s, *p* = 0.04), while SURG-TLX score (SLI: 88 vs. ALI: 69, *p* = 0.41) was comparable between both groups. Concerning the use of ALI between the SLI and the ALI group, task time (SLI: 600 s vs. ALI: 298 s, *p* < 0.001) and SURG-TLX scores (SLI: 166 vs. ALI: 79, *p* < 0.001) were differing significantly.

## Discussion

Articulating laparoscopic instruments (ALI) promise to improve laparoscopic dexterity and precision in challenging environments by providing additional degrees of freedom [3, 4]. However, evidence concerning their learning curve as well as performance in comparison to standard laparoscopic instruments (SLI) in controlled simulator environments remains limited.

Our study demonstrates that while ALI offer an additional degree of freedom that may conceptually enhance dexterity, their use was associated with longer task completion times, and less efficient motion patterns compared to SLI, particularly among participants without prior experience. Importantly, participants randomized to the ALI group demonstrated improved performance with ALI compared to the SLI group while showing similar performance in the use of SLI. This suggests that repeated training with ALI facilitates adaptation and performance for both SLI and ALI.

This study is the first that comprehensively evaluates dry lab learning curves and laparoscopic performance based on complex parameters which were first shown by Horemann and colleagues [22]. The ALI group was characterized by a prolonged learning curve based on several parameters such as task time, peak force and path length. This finding aligns with previous work highlighting the technical complexity of articulating instruments and their demand for higher cognitive and motor workload [2, 7, 23]. The increased SURG-TLX scores further substantiate that ALI impose greater mental and physical effort and is consistent with previous observations [2, 23].

Moreover, our study also demonstrated that trainees allocated to the ALI group achieved significantly better performance metrics when subsequently using ALI compared to the SLI group while SLI performance was in many assessed parameters not significantly different. This suggests that early exposure and targeted training with ALI may accelerate proficiency with both ALI and SLI. Preliminary evidence is also indicating a potential skill transfer from laparoscopic systems to robotic systems [24–27]. Due to the similarities of ALI with robotic systems, the skill transfer to robotic platforms could be even more pronounced although evidence supporting this potential skill transfer is currently lacking and should be explored in future studies. Considering these potential benefits, a prolonged learning curve when using ALI could be justified.

Our findings have important implications for surgical education. Given that clinical series have demonstrated the feasibility and safety of ALI across gastrointestinal and urological procedures, the use of ALI is more frequently observed in clinical settings [8–13]. Structured training curricula need to implement ALI to account for their unique handling characteristics and prolonged learning curve. Our results support the notion that exclusive training on SLI is insufficient preparation for ALI use. Instead, multimodal training curricula tailored to ALI may be necessary to ensure safe and efficient clinical translation [28].

## Limitations

Although the methodological quality of this study underlines the robustness of the data, several limitations need to be acknowledged. First, the study population consisted exclusively of medical students without prior laparoscopic experience. While this allowed for an unbiased evaluation of learning curves, the results may not generalize to surgical residents or experienced laparoscopic surgeons, who may adapt differently to ALI. Second, the study was conducted in a dry-lab setting using box trainers rather than in vivo or cadaveric models. Although this ensures standardization and objective measurement, it may not fully replicate the complexity of real tissue handling, haptic feedback, and intra-abdominal constraints. Third, the training period was relatively short and focused on basic tasks. A longer follow-up with more complex and also wet-lab procedures would provide deeper insights into long-term skill acquisition and retention. Fourth, although we employed validated force and motion analysis (ForceSense) and workload (SURG-TLX) measures, unmeasured cognitive factors such as spatial reasoning ability or psychomotor aptitude could have influenced individual performance trajectories. Fourth, although both the Peg transfer and the suture and knot tasks were trained and assessed throughout the curriculum, learning curves were analyzed only for the Peg transfer task due to the lack of baseline data for the suture and knot task. Consequently, the interpretation of learning curves is limited to the Peg transfer task and may not be generalizable to other tasks. Finally, the study was performed in a single-center and limited to one specific ALI system. Therefore, our findings may not be directly transferable to other articulating instrument platforms. In addition, due to the absence of preexisting data concerning the skill transfer from articulating to standard laparoscopic performance we were not able to conduct an adequate a priori power calculation. Therefore, our study could be underpowered for some of the performed analyses and results of this study must be interpreted with appropriate caution.

## Conclusion

Training with ALI has been shown to support skill acquisition with both ALI and SLI. While the use of ALI is associated with a prolonged learning curve, our results suggest a partial skill transfer to SLI. Considering the additional degrees of freedom provided by ALI and a potential and hypothetical skill transfer to robotic systems, their integration into minimally invasive training curricula may be of educational value for both students and surgeons.

## Supplementary Information

Below is the link to the electronic supplementary material.

Supplementary file1 (DOCX 138 KB)

Supplementary file2 (PPTX 248 KB)

Supplementary file3 (PPTX 381 KB)

Supplementary file4 (PPTX 311 KB)

## Abbreviations

ALI: Articulating laparoscopic instruments
AY: Academic year
DH: Dominant hand
FLS: Fundamentals of laparoscopic surgery
MIS: Minimally invasive surgery
NDH: Non-dominant hand
SLI: Standard laparoscopic instruments
