# Supplementary material for: Comparison of learning curves and cross-over effects using articulating laparoscopic instruments versus standard laparoscopic instruments
Source: Surg Endosc. 2026 Jun 19;40(8):6889–901. doi: 10.1007/s00464-026-12977-1 (PMC13457440; doi:10.1007/s00464-026-12977-1)
Supplement: Supplementary file 1 [file 464_2026_12977_MOESM1_ESM.docx]

**Supplementary Table 1:** Overview of the utilized error definition.

| **Task** | | **Error** | **Definition** | |
| --- | --- | --- | --- | --- |
| Peg transfer task | | 0 | No triangle dropped | |
|  | | 1 | Triangle(s) dropped | |
| Suture and knot task | |  |  | |
|  | Accuracy | 0 | Needle passes through both target points | |
|  | | 1 | Needle passes through only one target point | |
|  |  | 2 | Needle passes through neither target point | |
|  | Adaptation | 0 | Both edges of the Penrose drain touch |  |
|  |  | 1 | Edges approximate but do not touch |  |
|  |  | 2 | No visible approximation of the edges |  |
|  | Tightness | 0 | Knot holds securely, even under manipulation |  |
|  |  | 1 | Knot becomes loose upon manipulation |  |
|  |  | 2 | Knot is visibly loose without manipulation |  |
|  |  |  |  |  |

**Supplementary Table 2:** Comparison of error occurrence between the use of SLI and ALI for both the SLI group and the ALI group for the Peg transfer and the suture and knot tasks during the final exam and crossover assessment. Participants of the SLI group conducted the task using SLI and ALI. Likewise, participants of the ALI group conducted the task using both SLI and ALI

| **Task** | | **Error** | **SLI** | | **ALI** | **p-value** |
| --- | --- | --- | --- | --- | --- | --- |
| Peg transfer task | |  |  | |  |  |
| SLI group | |  |  | |  | **< 0.001** |
|  | | 0 | 22 (84.6) | | 7 (26.9) |  |
|  | | 1 | 4 (15.4) | | 19 (73.1) |  |
| ALI group | |  |  | |  | 0.148 |
|  |  | 0 | 15 (55.6) |  | 21 (77.8) |  |
|  |  | 1 | 12 (44.4) |  | 6 (22.2) |  |
| Suture and knot task | |  |  | |  |  |
| SLI group | |  |  | |  | **0.039** |
|  | Accuracy | 0 | 10 (38.4) | | 6 (23.1) |  |
|  | | 1 | 8 (30.8) | | 3 (11.5) |  |
|  |  | 2 | 8 (30.8) | | 17 (65.4) |  |
|  | Adaptation |  |  |  | | **0.001** |
|  |  | 0 | 23 (88.5) | 11 (42.3) | |  |
|  |  | 1 | 3 (11.5) | 7 (26.9) | |  |
|  |  | 2 | 0 (0) | 8 (30.8) | |  |
|  | Tightness |  |  |  | | **0.004** |
|  |  | 0 | 24 (92.3) | 14 (53.8) | |  |
|  |  | 1 | 2 (7.7) | 4 (15.4) | |  |
|  |  | 2 | 0 (0) | 8 (30.8) | |  |
| ALI group | |  |  |  | |  |
|  | Accuracy |  |  |  |  | 0.943 |
|  |  | 0 | 6 (22.2) |  | 7 (25.9) |  |
|  | | 1 | 8 (29.6) | | 8 (29.6) |  |
|  |  | 2 | 13 (48.2) | | 12 (44.5) |  |
|  | Adaptation |  |  |  | | 0.454 |
|  |  | 0 | 22 (82.5) | 20 (74.1) | |  |
|  |  | 1 | 3 (11.1) | 2 (7.4) | |  |
|  |  | 2 | 2 (7.4) | 5 (18.5) | |  |
|  | Tightness |  |  |  | | 0.236 |
|  |  | 0 | 26 (96.3) | 23 (85.2) | |  |
|  |  | 1 | 0 (0) | 3 (11.1) | |  |
|  |  | 2 | 1 (3.7) | 1 (3.7) | |  |
|  |  |  |  |  | |  |
